# The Essence of Systems Chemistry

**DOI:** 10.3390/life9030060

**Published:** 2019-07-11

**Authors:** Peter Strazewski

**Affiliations:** Institut de Chimie et Biochimie Moléculaires et Supramoléculaires (Unité Mixte de Recherche 5246), Université de Lyon, Claude Bernard Lyon 1, 43 bvd du 11 Novembre 1918, 69622 Villeurbanne CEDEX, France; strazewski@univ-lyon1.fr; Tel.: +33-472-448-234

**Keywords:** human intervention, origin of life, metaphors, science communication

## Abstract

Systems Chemistry investigates the upkeep of specific interactions of an exceptionally broad choice of objects over longer periods of time than the average time of existence of the objects themselves. This maintenance of a dynamic state focuses on conditions where the objects are thermodynamically not very stable and should be rare or virtually inexistent. It does not matter whether they are homochirally enriched populations of chiral molecules, a specific composition of some sort of aggregate, supramolecules, or even a set of chemically relatively unstable molecules that constantly transform one into another. What does matter is that these specific interactions prevail in complex mixtures and eventually grow in numbers and frequency through the enhancing action of autocatalysis, which makes such systems ultimately resemble living cells and interacting living populations. Such chemical systems need to be correctly understood, but also intuitively described. They may be so complex that metaphors become practically more important, as a means of communication, than the precise and correct technical description of chemical models and complex molecular or supramolecular relations. This puts systems chemists on a tightrope walk of science communication, between the complex reality and an imaginative model world. This essay addresses, both, scientists who would like to read “A Brief History of Systems Chemistry”, that is, about its “essence”, and systems chemists who work with and communicate complex life-like chemical systems. I illustrate for the external reader a light mantra, that I call “to make more of it”, and I charily draw systems chemists to reflect upon the fact that chemists are not always good at drawing a clear line between a model and “the reality”: The real thing. We are in a constant danger of taking metaphors for real. Yet in real life, we do know very well that we cannot smoke with Magritte’s pipe, don’t we?

The pioneering march towards the vast and fruitful lands of Systems Chemistry made its start in 1986 with Günter von Kiedrowski’s first experimental discovery of the autocatalytic self-replication of a self-complementary hexadeoxyribonucleotide, being ligation product and template in one. Its concentration grew from a chemical system that was fed with two synthetically end-capped trinucleotides and fueled with a well-working water-soluble coupling agent, ethyl-3-(3-dimethylaminopropyl) carbodiimide (EDC), which could only activate the starting material and not the product [1]. More astonishing than the fact that this chemical ligation proceeded enzyme-free was the ligation kinetics. A seminal follow-up from similar but non-palindromic ligation products showed that cross-catalysis of non-self-complementary templates would progress in the same parabolic growth regime as autocatalysis [2]. The “square-root law” was born, and with it the most pertinent scientific questions on the population dynamics of “simple” replicators have been thrown up that are still being asked today [1,2,3,4,5,6].

After twenty years of pregnancy—a long, seemingly inert lag phase is characteristic for autocatalytic processes—Systems Chemistry, begotten from mother Bioorganic and father Prebiotic, was baptized by its founder GvK. Already, the first workshop on Systems Chemistry in Venice 2005 revealed that the research in this area would become very wide, as well as very innovative, and bore a huge potential for young chemists [7]. The childhood and adolescence of Systems Chemistry was not very well tempered (sometimes still isn’t), as you would expect from the youth. Its close family, and certain more remote family members, who in one way or another were the forerunners of his arrival, hailed Systems Chemistry right from the start [8]: Sister Homochirality, the twins Composome and Dynamic-Kinetic, uncle Metabolism, auntie Compartment, and of course ever-present grandma Evolutionary-Genetics. Only grandpa Synthesis was shrugging his shoulders, too old to see what’s new in the uprise of his late grandchild. Soon, second- and third-degree cousins voiced out: Cousin Artificial-Intelligence, cousin Philosophy, and cousin Cosmology. In all of these vivid and highly creative discussions, it was always father Prebiotic, backed by cousin Philosophy, who tried to have the last word [9]. But, of course, this never works, not in a badly-behaved discipline like Chemistry—no manners. Then, out of the blue, Systems Chemistry got married to a foreigner—of course a foreigner, no incest is tolerated in this family! Her maiden name is Supramolecular, sounds lovely, well dressed in a Nobel Prize, too. Actually, Miss Supramolecular was not exactly supramolecular in the classical sense [10,11], since her main aim was to get untied from the Flying-Pig branch of her family, as is often the case in these young marriages. She, too, has widespread ancestors to tell about, like extravagant auntie SpatioTemporal, the far-out twins Nano-Tech and Single-Molecule, or her youngest sister Nobel-Motion, to name the most prominent. Thus far, there is no evident explicit progeny from this still very young couple but, just in case, they have invited wet nurse Ms. BZ (Belousov–Zhabotinsky in full), and other members of that guild, to escort their further endeavor [12]. This was in full accord with the parents of Systems Chemistry, and of course a great move! The latest rumor is that Systems Chemistry and Supramolecular are in expectancy after all. If it’s a girl, her name will be Kinetic Asymmetry [13], or if it’s a boy, Kay Ratchet [14]. Do you like metaphoric storytelling? No? I love it! I am a chemist. As recently addressed by Michelle Francl in “it’s alive!”, chemists are extremely fond of metaphors, especially while teaching [15].

When Bachelor students ask me “what’s systems chemistry?”, “c’est quoi la chimie systémique?”, I usually reply first with a counter question and a metaphor, which goes like this: Who do you think discovered the first chemical reaction? [shrugging] Since chemistry, unlike physics and biology, is a non-historical science, I cannot really know for certain. However, I do imagine that it happened a very long time ago, perhaps even before the agricultural revolution, and close to a fire (activation energy) where lots of ashes were present (strongly alkaline chemicals), some water too, and food needed to be cooked. I would like to think about this first chemist as being a lady. It seems plausible for some of us that in hunter–gatherer communities, usually the gentlemen were hunting and gathering, while the ladies stayed close to the caves and houses, looked after the smaller children, and took care of the hunted and gathered. Perhaps through serendipity, as so often is the case in science, a big piece of wet fat fell unnoticed into the fire on the ashes, which might have contained lots of sodium and potassium salts, maybe from sea water? After some time, the fat transformed into a soft solid (soap), which precipitated from a clear liquid (glycerol). If I’d been in the place of this lady, I would have been quite astonished, and the first thing I’d do after the stuff cooled down, I’d probably dip my dirty fingers into this soapy mass, I’d smell it (hm), and taste it—yerk! I’d perhaps panic a bit and smear some of the stuff over my face out of disgrace. I’d immediately look for some freshwater to get rid of it, and hey presto—what an effect! I’d be even more speechless than before. Lucy might have thought about this useful effect (clean hands), even more than about what it actually was that produced this soap, viz. the first chemical reaction under kind of controlled and reproducible conditions—today we call it an ester hydrolysis under alkaline reaction conditions, a saponification. Let us assume that the first chemists, as today, usually belonged to middle-class clans, and at the time, mirrors were luxury objects only to be dreamt of. Lucy’s family members would have noticed this effect even more than herself. Humans have always been very social animals, but also ambitious, and therefore jealous. Lucy’s clean face would not stay unnoticed by the first chemist’s comrades, for sure. Her sister immediately wanted to have more of it (Figure 1A). Eager to look clean and attractive, she would convince her best mates to hunt for more of this fat, since the sisters were in a hurry to optimize conditions for an upscaling process in their laboratory. Thus, Chemistry as a science was born and almost instantaneously with it, Systems Chemistry (Figure 1B). 

It is very likely that these primordial scientific disciplines eventually went unnoticed for a very long time but, when you look at it in retrospective, Chemistry and Systems Chemistry have always been a nice pair. Chemistry is the science that produces and isolates new and pure substances from controlled chemical reactions. These procedures require highly knowledgeable human intervention. Systems Chemistry is the discipline that studies the mechanisms of the dynamic upkeep of chemical substances. The main focus is the maintenance of those particular substances that would rapidly and almost completely degrade without the conserving mechanisms. The main challenge here is to find the right initial conditions for the upkeep effect to prevail “on its own”, yet very often while supplying the system with energy, for a maximally long period of time. Both disciplines have in common that any useful effect of a chemical reaction can become *a seed for a network* of chemical reactions that may provide *more of it*. A subtle difference between plain Chemistry and Systems Chemistry is that the researcher seeks more to back off from the latter than from the former, tries not to be the central scrutinizer nor the intelligent designer, and does not want to interfere once the system works, not more than absolutely necessary [16]. An ideal chemical system is “left on its own while it grows”. Another important difference is the focus—traditional chemists concentrate on the substances, while systems chemists ponder on the dynamic interactions between different substances. 

In essence, Systems Chemistry is the study of chemical reaction conditions that provide synergies to make more of the stuff that is useful for the whole system [17]. I hear a few colleagues who naturally oppose: What is “useful”, who or what “makes more”, what do you mean by “it”? Think generously, something is apparently “useful” when it hangs around for a long time and persists against the odds. Ideally, nobody “makes more”—the scientist should merely set the initial conditions and let the system self-evolve into making “more of it” on its own. The “it” can be almost anything. For example, “it” could be chirally more and more homogenous molecules or supramolecular complexes that persist longer and become gradually enriched from weakly scalemic mixtures. “It” could be a supramolecular association (micelles, piles, fibers) that grows in numbers, and thus, replicates its chemical composition or/and (and!) moves anisotropically (molecular robot) under the control of fuel addition–depletion and waste disposal. “It” could be a whole proto-metabolic reaction network that is more frequent, more abundant, robust, and at high turnover despite all side reactions and parasitic cycles that happen to be connected with the useful cycles, always menacing to drain them. “It” could be a population of semipermeable micro-reactors containing selectively confined macromolecular reactants, filled compartments, for instance, that persist longer under stressful (selective) conditions because they grow and divide (replicate) faster, and self-evolve upon feeding and fueling, to become more robust than other micro-reactors that compete for the same resources. At the highest level of complexification, we are looking at biological life. The complexity of the most primitive micro-organisms is so flabbergasting, when compared to any chemical system, that we can only resort to metaphors, when a non-specialist asks us “what is life?” [18]. Irrespective of metaphoric language and descriptions, somehow real life must have started, musn’t it? Somehow, we scientists should be able to explain it, shouldn’t we (Figure 2)?

In situations where in-depth explanations are out of place, metaphors are the ideal means for painting lively pictures that “speak to the listener”. However, there is also a hidden side of metaphors—no dark side (of the moon) of course—so let me briefly navigate out there and take a closer look [19]. Rather than elaborate on the use of conceptual metaphors by comparing statements presented here with published work on science communication, such as Theodore Brown’s “Making Truth: Metaphor in Science” [20], I prefer to carry on with my personal impression over the years of the development of Systems Chemistry [8,12]. In retrospective, the most challenging task during the beginning phase of Systems Chemistry was to find a common and maximally precise language for the whole interested community across disciplines like organic chemistry, evolutionary biology, information theory, cosmological physics, philosophy, and so forth, including the respective relevant sub-disciplines. Seemingly straight-forward notions, such as energy, information, communication, cooperation, dynamic, kinetic, function, selection, error propagation, purpose, replication, reproduction, and evolution have similar but distinctively different meanings across these disciplines and sub-disciplines. Much effort needs to be committed (work under permanent construction), in order to set, as well as possible, standards for a common language to explain to one another how best should be defined and understood this or that notion. For example, when we say, “Darwinian evolution”, we mean a change in frequency and abundance of a heritable trait of a population, through adaptation and in competition (“fitness”) with others, as opposed to through random drift, migration, or molecular changes per se. It applies to populations of entities capable of multiplication, variation, and heredity. There is nothing purely biological in this definition—the term “population” is one that we use in statistics: An ensemble of entities. “Genetic” algorithms realize an evolving system, and there is nothing living about them, not in the conventional sense of the word [21,22,23]. Darwinian evolution can thus apply to living or non-living entities, genetic algorithms, or viruses. Another example: To call a chemical process, that produces peptides from amino acids catalyzed by ribonucleotides, a “translation system” may be misleading, unless information encoded in one polymer is actually, and more or less reliably, translated into the other polymer. There is no translation happening in a mere peptidyl transfer from one nucleotide to another, as difficult as it is at the moment to realize this by experiment. Yet another: The word replication stems from cloned bacterial “replica”, but is used for identical copies of almost anything that grows in numbers. Apart from classical replicators, such as bacterial clones and auto/cross-catalytic templating covalent macromolecules (vide supra), we know that emulsion droplets, coacervates, and giant lipid vesicles are able to grow in size and divide in smaller similar objects, thus, to replicate their usually spherical shapes, if not their molecular composition [24]. Furthermore, supramolecular Systems Chemistry is growing fast and shows how non-covalent assemblies of designed objects replicate their shape and identity [10,11,17]. There is much chemistry out there where the “making more of it” is waiting to be discovered. But to call such systems alive right away would not be scientifically responsible. 

When could we call designed constructs alive; for example, self-repairing robots? Note that to “re-pair” could mean to re-make one functional whole out of two dysfunctional parts. Show me a robot that self-reproduces by somehow giving birth to at least one similar, also self-reproducible daughter robot—hence, a robot that makes at least two out of one instead—and I will be happy to call her alive. However, complex chemical sub-systems that bear separate or coupled implementations of “replication”, “metabolism”, “containment”, and any other model or metaphor (cf. the “Los Alamos bug”) just cannot be seriously called “alive” or “living”, or else we would degrade our own credibility. This is the hidden side of metaphors, this is where Systems Chemistry needs to tame its language. Maybe in the future we will be able to create truly living chemical systems. For this to happen, we need exponentially replicating robust entities containing information carriers that are *highly variable in composition*—primary sequences or other stores of readable information—and can transfer this highly diverse “genetic” (inherited, readable) information from one generation to another within an error margin that sets a useful limit to its total information content to be replicated, in order to thrive towards *open-ended evolution*, to self-evolve, and thus be alive, living, animate. Spherical or rod-like shapes of uniform and unchanging composition that exponentially self- or cross-replicate over and over again, or exponentially replicating ribozymes of a specific and hardly modifiable composition, do not quite deserve this attribute. They are models, metaphors, goals crucial for our deeper understanding, but they are not alive. 

When cosmologists tell us about the “birth of stars”, the “big bang”, and that “black holes have no hair”, nobody thinks that they might actually confuse their models with the reality. A cosmologist or experimental particle physicist will not desire—perhaps only in his or her wildest dreams—to create a real, not in silico, “synthetic Universe”. Given our fervor for an early “inflationary”, sudden cosmic expansion of the total space volume over at least twenty-six orders of magnitude, it may be too dangerous to do so at this stage of research. There are physicists that would like to produce, in a particle accelerator, small experimental mini-black holes, which should be very short living, if feasible at all. Usually, cosmologists and particle physicists hardly ever confuse a model with the reality; too different are the looks of both. Things look different on a less energetic scale of experimentation. Still, the difference between “electron-loving” and “atom nucleus-loving” (electrophilic and nucleophilic) molecular “attacks” on the one hand, and our young mothers and fathers on the other hand, is too large to be confused; so are the differences between us and “electron-hungry” atoms, “leaving” or “functional” groups, “water-hating” molecules, “breathing” double-strands, or even “living” dynamic combinatorial libraries. The difference between the looks and behavior of Dr. Frankenstein’s childless monster and yours or mine—well, my looks for sure—is too large to be confused by any biologist, I hope. 

The essence of Systems Chemistry is to explore the chemical space of appropriate initial conditions and energy supplies for chemical mixtures to maintain a dynamic state of chemical substances that spontaneously grow in numbers as time goes by. The main methodological challenge to do so is to minimize human intervention after its initiation. The technical novelty, when compared to traditional approaches in chemistry, is the analytical handling of complex mixtures without necessarily separating them. The ground-breaking transition for the field of chemistry is the deep understanding of the origins of biological complexity. The sought-for next “quantum leap” is to generate self-evolving properties, which themselves generate self-evolving behavior. The “Holy Grail” is to create new life from inert matter. 

The essence of this essay on Systems Chemistry is to raise the finger and say: “Watch it, folks!” (Figure 3), the closer to life our chemical systems are in their complexity, the more careful we should use our language to describe them. This is a dilemma that we need to be increasingly aware of, not to abuse the communication of metaphors. Or else, sloppy and over-enthusiastic metaphoric language, not to say blunt lies, will make “them” think that the next thing we will do is to actually try to smoke with Magritte’s painted pipe. This would not be a good future for Systems Chemistry (to be continued elsewhere).

## Figures and Tables

**Figure 1:**
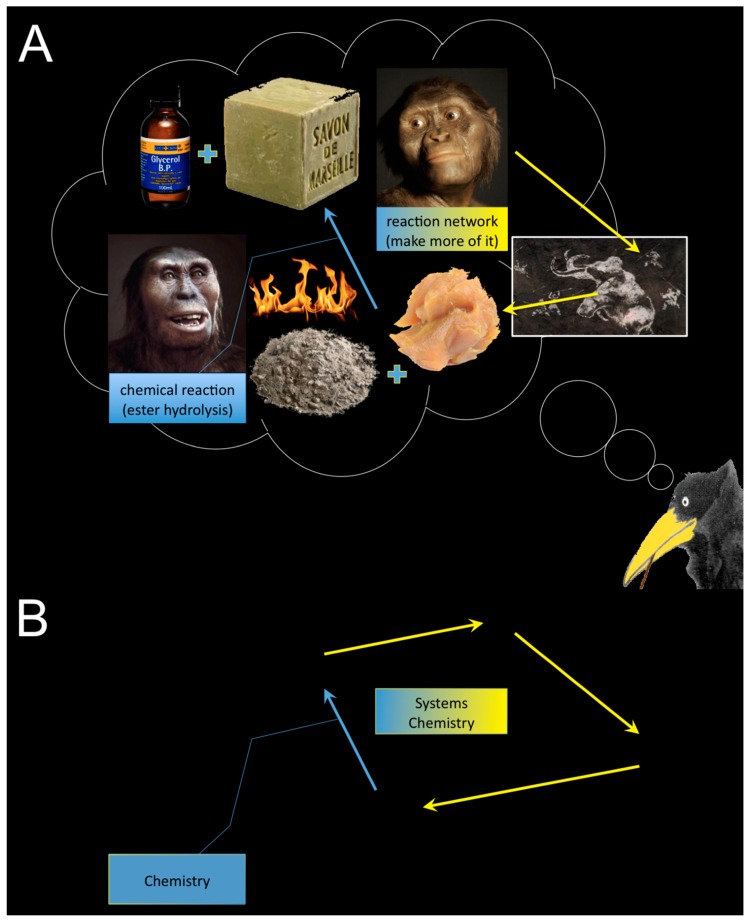
(**A**) The invention of the first chemical reaction (hypothesis) and (**B**) its relation to Systems Chemistry: “to make more of it”, cf. explanation in the text above.

**Figure 2:**
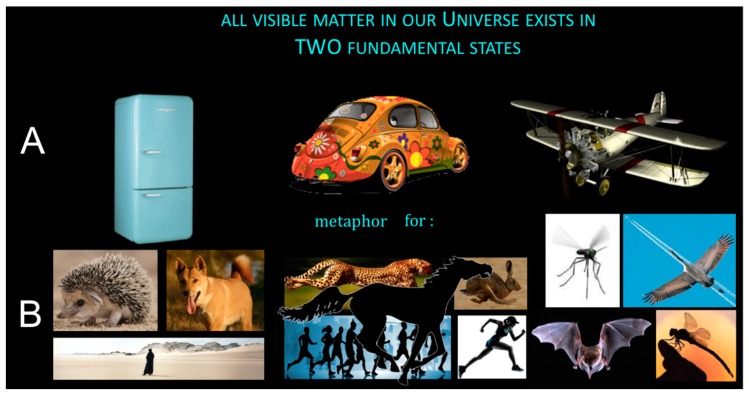
(**A**) Inert (inanimate) state of matter: Reproducible and evolvable objects that can be evolved by an external designer for useful functions. (**B**) Alive (animate) state of matter: Self-reproducing self-evolvable organisms perform the same useful functions. Columns 1, 2, 3, respectively, left: Thermostasis (fridge versus warm-blooded animal: Desert hedgehog, dingo, human in desert); middle: 2D-motion (car versus rapid animal: Cheetah, hare, horse, running people); right: 3D-aero motion (engine aeroplane versus actively flying animal: Mosquito, stork, bat, dragonfly). The functions in (**A**) may be useful for the external designer to persist comfortably, there is no purpose (function) for the object in itself. The functions in (**B**) may be useful for the organism itself to persist through reproduction. Objects in (**A**) are seen as (inert) metaphors for organisms in (**B**) [19]. Reproduced with permission from © Taylor & Francis, 2019.

**Figure 3:**
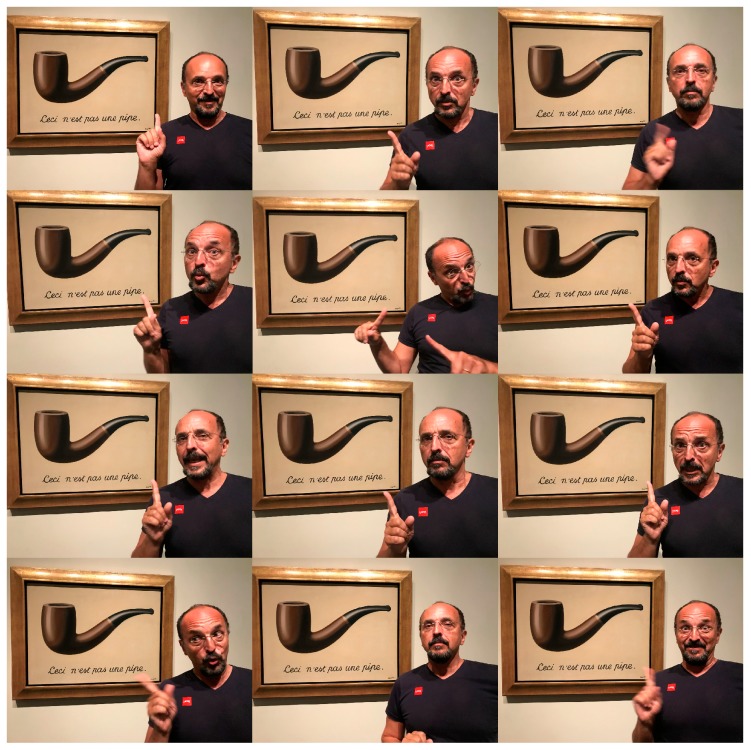
Picture collage of the author on 24 August 2018 in front of René Magritte’s original painting exposed in the Los Angeles County Museum of Arts (LACMA) © ADAGP, Paris, 2019.
